# Ovarian dysfunction in adolescent girls with autoimmune rheumatic diseases

**DOI:** 10.1186/s12969-023-00923-7

**Published:** 2023-11-14

**Authors:** Mohamed Taman, El-Hadidy NM, Hamed Yousef, Doaa Mosad Mosa

**Affiliations:** 1 Assistant professor of Obstetrics & Gynecology, Faculty of Medicine, Mansoura University Hospitals, Mansoura University, Mansoura, Egypt; 2 Department of Public Health and Community Medicine, Faculty of Medicine, Mansoura University, Mansoura, Egypt; 3 Professor of Obstetrics & Gynecology, Faculty of Medicine, Mansoura University Hospitals, Mansoura University, Mansoura, Egypt; 4 Lecturer of Physical medicine, Rheumatology and Rehabilitation, Mansoura University Hospitals, Mansoura University Faculty of Medicine, Mansoura, Egypt

**Keywords:** Systemic autoimmune rheumatic Diseases, Menstrual abnormalities, Puberty, Gonadal dysfunction

## Abstract

**Objective:**

To explore the different menstrual and pubertal abnormalities in adolescent females with systemic autoimmune rheumatic diseases (ARD).

**Methods:**

The study included adolescent girls aged 13–18 years with juvenile idiopathic arthritis (JIA), juvenile systemic lupus erythematosus (JSLE), and juvenile dermatomyositis (JDM) classified according to their international classification criteria. Data were collected from our patients’ files and interpreted with respect to the demographic, clinical, disease assessment parameters, medications used, and the hormonal profile. The aspects of puberty and menstruation were assessed by a gynaecologist with ultrasound evaluation as well. The girls were classified according to their menstrual pattern into those with regular cycles versus abnormal ones. The subgroups were compared and significant variables entered into a logistic regression model to detect the independent predictors.

**Results:**

Twenty-one girls with JSLE were included, besides 23 JIA and 8 JDM cases. Ten patients with JSLE (47.6%) had menstrual abnormalities, whereas only four JIA (17.4%) and 1 JDM girls had these alterations without significant difference between the three groups. The median of the SLICC/ACR damage index was statistically higher in JSLE with abnormal menstrual cycles, similarly were the cumulative steroid dose and puberty onset. No difference was observed between JIA or JDM subgroups concerning the disease parameters, hormonal profile, ultrasound assessment or the treatment lines. The most significant predictor for menstrual abnormalities in JSLE was the SLICC/ACR damage index.

**Conclusion:**

Menstrual abnormalities is a common disturbance among adolescent girls with ARDs. The SLICC/ACR damage index is the main determinant for menstrual abnormalities rather than the cumulative steroid use or disease duration in JSLE.

## Highlights

JSLE cases had a higher rate of menstrual abnormalities more than JIA or JDM girls.

The damage index was statistically higher in JSLE with abnormal menstrual cycles, similarly were the cumulative steroid dose and puberty onset.

The most significant predictor for menstrual abnormalities in JSLE was the SLICC/ACR damage index as inferred from the regression analysis.

## Introduction

Autoimmune diseases are a wide range of disorders in which the immune system attacks self-antigens resulting in damage or dysfunction of the tissues. During puberty, autoimmune rheumatic diseases (ARD) are more common in females than in males like juvenile idiopathic arthritis (JIA), juvenile systemic lupus erythematosus (JSLE), and juvenile dermatomyositis (JDM), which may derail the normal stages of puberty or menstruation [1].

Puberty is the acquisition of secondary sex characteristics that associated with the growth spurt, resulting in the accomplishment of reproductive function and final height [2]. The presence of one or more of the following clinical and laboratory parameters defined normal gonadal function: normal menstrual cycles with or without dysmenorrhea, elevated cervical mucus length, normal levels of plasma follicular stimulating hormone (FSH), luteinizing hormone (LH), estradiol (E2), progesterone, prolactin, and testosterone. In addition to the normal urinary hormonal cytology or serial pelvic ultrasound (US) compatible with ovulatory pattern [3].

Disorders of puberty can profoundly impact the physical and psychosocial well-being. Precocious puberty is the pubertal onset before eight years of age in girls, while delayed puberty is defined as the outset of pubertal changes was delayed more than 13 years in girls, or age of menarche > 16 years [4].

The gonadal function alternation may be mediated by several factors that related to the adolescence and ARD, such as physiologic anovulation of adolescence, dysfunction of the hypothalamic–pituitary–ovarian axis, thyroid disorders, and the immunosuppressant agents used, particularly cyclophosphamide and corticosteroids [5–7].

Health care providers who counselling adolescents with juvenile ARDs need to take in consideration of these problems to minimize the negative impact of ARDs and their medications. Patients and families will benefit from the increased awareness of the relationship between ARDs and ovarian dysfunction, menstrual abnormalities, or puberty disorders.

Studies that addressed the impact of ARDs on ovarian function or puberty-related outcome are scare. This study aimed to shed light on the different menstrual and pubertal abnormalities observed in young females with systemic ARDs, and its relation to the various disease parameters.

## Patients and method

This cross-sectional study was conducted on a group of adolescent girls with ARDs attending to the Paediatric Rheumatology Clinic, Mansoura University Children’s Hospital. The study was conducted from October 2021 till December 2022 and all eligible cases were collected along this duration according to the inclusion criteria.

### Inclusion criteria

Adolescent girls aged 13–18 years with the following ARDs:

- JIA cases who are classified based on the International League of Associations for Rheumatology (ILAR) criteria [8].
- JSLE patients who are classified according to the 2019 European League Against Rheumatism/American College of Rheumatology (EULAR/ACR) Classification Criteria for Systemic Lupus Erythematosus [9].
- JDM girls who are classified on the basis of the EULAR/ACR classification criteria for juvenile idiopathic inflammatory myopathies and their major subgroups [10].

### Exclusion criteria

1. Patients with other chronic diseases (endocrinal, reproductive, neurological, or cardiac), which may affect the pubertal maturity or regular menstruation.
2. Females using hormonal contraceptive agents.
3. Patients/ parents refuse to share in this special research.

### Work plan

Data were collected from our patients’ files and interpreted with respect to the demographic, clinical, disease assessment parameters, medications used, and the hormonal profile as follows:

1. Disease assessment indices at the time of enrollment including: disease activity by juvenile arthritis disease activity score-10 (JADAS-10) [11], disease damage by juvenile arthritis damage (JAD) index [12] in case of JIA, while in JSLE cases; the disease activity and cumulative damage were measured using the SLE Disease Activity Index 2000 (SLEDAI-2 K) [13] and the Systemic Lupus International Collaborating Clinics/ACR (SLICC/ACR) Damage Index, respectively [14]. In cases with JDM, Myositis disease activity assessment tool (MDAAT) was used [15].
2. Laboratory investigations: Complete blood count (CBC), erythrocyte sedimentation rate (ESR), C-reactive protein (CRP), liver function tests, serum creatinine, complement, urine analysis, muscle enzymes, and autoimmune profile (anti-nuclear antibody (ANA), rheumatoid factor (RF), Anti-dsDNA) as routine laboratory tests in caring for cases with ARDs. The determination of serum hormonal levels was at the follicular phase (between day 2 and day 7 of the menstrual cycle or randomly for girls with amenorrhoea), including E2 (normal range: 22–215 pg/mL), LH (1.1–11.6 mIU/L), FSH (3–14.4 mIU/L), and prolactin (1.9–25 ng/mL) [16].

The following aspects of puberty were assessed: the age at puberty onset, age at menarche, menstrual abnormalities, and the length of menstrual flow. Each patient was examined by a gynaecologist for secondary sexual characteristics according to the Marshall and Tanner’s pattern of pubertal changes [17] then they were specified according to the breast maturity into B1–B5 and pubic hair maturity into PH1– PH5. Pubertal onset is defined as the onset of B2/PH2, meanwhile delayed puberty is identified as the age at B2/PH2 > 13 years or onset of menarche > 16 years [18].

The normal cycle was identified as having a cycle length varying from 28 to 35 days, with 3–7 days of blood flow. Menstrual irregularities were based on alterations in one or more of these parameters. While, amenorrhoea and sustained amenorrhoea is defined as the cessation of menstruation for more than 4 months after menarche and persisting for more than 12 months, respectively [3, 16].

US assessment was performed using a 7.5 MHz scan at the follicular phase (2nd or 3rd day of menstruation). The evaluated US parameters were the diameter of endometrium (normal 6–10 mm) and ovarian volumes (normal 8–10cm3) [3].

### Statistical analysis

Data were analysed using the IBM-Statistical Package of Social Science (SPSS) program for Windows (version 22). The normality of data was first tested. Qualitative data were described using numbers and percentages. Continuous variables were presented as mean ± standard deviation (SD) for normally distributed data and median (min-max) for non-normal data. The comparison between the three ARDs was performed with the Kruskal-Wallis test while the Mann Whitney test was used between those with menstrual abnormalities versus those with regular cycle within each ARD.

The comparison between the categorical variables was tested using the Chi-square test while the Fischer exact test was used when the expected cell count less than 5. The Spearmen correlation was utilized to detect the association between the age of menarche, disease duration, activity, and the cumulative doses of steroid in girls with ARDs. The logistic regression model was performed to predict the most significant determinants for menstrual abnormalities. The odds ratio (OR) was calculated with 95% confidence intervals. The results were considered significant when p ≤ 0.05.

## Results

### The demographic features and BMI of participants

Twenty-one adolescent girls with JSLE were included, the median of their current age, the age of disease onset, and the duration of disease were 16, 13, and 2 years, respectively. Their body mass index (BMI) ranged between 18 and 30 kg/m2. Meanwhile, twenty-three girls were enrolled with JIA (8 cases with oligo-articular subtype, 10 patients with poly-articular JIA, 3, and 2 children had enthesitis-related arthritis, and systemic JIA, respectively). The median of their current age was 14 years. The median of their age at the disease onset and disease duration were 11 and 4 years, respectively. Their BMI was between 17 and 32 kg/m2. The demographic features of JDM cases are shown on Table 1. The disease onset, diagnosis, and the disease duration were significantly different between JSLE and the other groups.

**Table 1:** Demographic features, disease onset, diagnosis, duration, and clinical features among adolescent girls with autoimmune rheumatic diseases

	Adolescent girls with autoimmune rheumatic diseases			
	Total (n = 52)	JSLE (n = 21)	JIA (n = 23)	JDM (n = 8)
	Median (Minimum to Maximum)			
Current age (years)	15 (13-17.5)	16 (14-17.5)	14 (13–17)	15 (14-16.5)
BMI	22 (17–32)	21.5 (18–30)	23.7 (17–32)	22 (18–23)
Age of disease onset (years) *#	12 (4–16)	13 (8–16)	11 (4-14.5)	9.5 (8–11)
Age of disease diagnosis (years) *	12.5 (5–16)	13 (7–16)	11 (5–15)	10 (10–13)
Duration of disease (years) *#*@*	3 (0.2–10)	2 (0.2-5)	4 (1–10)	5.8 (4–7)
Clinical features N (%)				
Fever	9 (17.3)	5 (23.8)	3 (13.0)	1 (12.5)
Weight loss	4 (7.7)	4 (19.0)	0 (0.0)	0 (0.0)
Fatigue	22 (42.3)	17 (81.0)	3(13.0)	2(25.0)
Lymphadenopathy	1 (1.9)	1 (4.8)	0 (0.0)	0 (0.0)
Mucocutaneous	15 (28.8)	12(57.1)	0 (0.0)	3(37.5)
Eye disease	0 (0.0)	0 (0.0)	0 (0.0)	0 (0.0)
Myositis	6 (11.5)	3(14.3)	1(4.3)	2(25.0)
Cardiopulmonary	5 (9.6)	4(19.0)	0 (0.0)	1(12.5)
GIT	1 (1.9)	0 (0.0)	1(4.3)	0 (0.0)
CNS	3 (5.8)	3(14.3)	0 (0.0)	0 (0.0)
Renal	6 (11.5)	6(28.6)	0 (0.0)	0 (0.0)
Arthritis/arthralgia	42 (80.8)	19 (90.5)	20 (87.0)	3(37.5)

BMI: body mass index, corresponding symbols indicate significant difference between groups; *: Significant difference between JSLE and JIA, #: Significant difference between JSLE and JDM, @: Significant difference between JIA and JDM

### The clinical features, disease activity, damage indices, and the treatment of ARD

The frequencies of clinical presentations of these adolescent girls were summarized on Table 1 and the most common reported manifestation among all diseases was arthralgia/arthritis. The disease activity, damage parameters, and physician assessment of disease activity states for JSLE, JIA, and JDM were displayed on Table 2. Concerning the lines of management, they varied according to the disease and its severity ranging between steroid (oral or pulses), methotrexate, hydroxycholoroquine, azathioprine, mycophenolate mofetil, or intravenous cyclophosphamide (CYC).

**Table 2:** Disease activity, damage indices, and physician assessment of disease states among adolescent girls with autoimmune rheumatic diseases

Types		Disease indices			Adolescent girls with autoimmune rheumatic diseases (n = 52) Median (Minimum to Maximum)		
JSLE		SLEDAI-2 K			4 (2 to 25)		
		SLICC/ACR damage index			2 (0 to 4)		
JIA		JADAS-10			5 (0 to 33)		
		JAD-index			0 (0 to 2)		
JDM		MYOACT			12 (0 to 30)		
		MITAX			5 (0 to 30)		
N (%)							
			Total (n = 52)	JSLE (n = 21)	JIA (n = 23)	JDM (n = 8)	
Physician assessment of disease activity states	High disease activity		7 (13.5)	3 (14.3)	3 (13.0)	1 (12.5)	Montecarlo exact test, *p*_1_ = 0.090 *p*_2_ = 0.115 ***p***_**3**_ = 0.0358*
	Moderate disease activity		11 (21.2)	6 (28.6)	3 (13.0)	2 (25.0)	
	Low disease activity		13 (25.0)	6 (28.6)	3 (13.0)	4 (50.0)	
	Remission on medication		20 (38.5)	6 (28.6)	14 (60.9)	0 (0.0)	
	Remission off medication		1 (1.9)	0 (0.0)	0 (0.0)	1 (12.5)	

SLEDAI-2 K: Systemic lupus erythematous disease activity index, SLICC/ACR: Systemic Lupus International Collaborating Clinics/ACR damage index, JADAS-10: juvenile arthritis disease activity score-10, JAD-index: juvenile arthritis damage, MYOACT: Myositis disease activity assessment visual analogue scales, MITAX: Myositis intention to treat activity index, p1: Significance between JSLE and JIA, p2: Significance between JSLE and JDM, p3: Significance between JIA and JDM. * P values ≤ 0.05 is considered statistically significant

### The pattern of menstrual and pubertal changes of ARD

The median age of menarche for patients with JSLE was similar to JIA and JDM groups and it occurred around the age of 12 years. The median of the menstrual blood flow was the same in 3 groups (5 days). The frequency of dysmenorrhoea was 33.3% and 34.8% in JSLE and JIA, respectively. Ten patients with JSLE (47.6%) had menstrual abnormalities (primary amenorrhea (3 cases), polymenorrhea (2), and oligomenorrhea (5)), whereas only four JIA (17.4%) and 1 JDM girls had these alterations without significant difference between the three groups regarding the pattern of menstrual changes (Table 3). Delayed menarche was found in 4 patients (8.5%); 3 of them with JSLE.

**Table 3:** Pattern of puberty and menstrual changes among adolescent girls with autoimmune rheumatic diseases

	Total (n = 52)	JSLE (n = 21)	JIA (n = 23)	JDM (n = 8)
Age at menarche (years)	12 (0–17)	12 (0–17)	12 (11–14)	12 (0–13)
Length of menstrual flow	5 (0–9)	5 (0–8)	4 (1–9)	5 (0–6)
Age at puberty onset	12 (0–15)	12 (0–15)	12 (11–14)	12 (0–13)
Tanner stage of breast	4 (1–4)	4 (1–4)	4 (1–4)	4 (2–4)
Tanner stage of pubic hair	3 (0–4)	3 (0–4)	4 (1–4)	3 (0–4)
Tanner stage of axillary hair	3 (0–4)	3 (0–4)	4 (1–4)	3 (0–3)
	N (%)			
Delayed puberty	6 (11.5)	3 (14.2)	2 (8.6)	1 (12.5)
Delayed menarche	4 (8.5)	3 (15.0)	0	1 (12.5)
Menstrual irregularities	15 (28.8)	10 (47.6)	4 (17.4)	1 (12.5)
* Primary amenorrhea*	*4*	*3*	*0*	*1*
* Polymenorrhea*	*2*	*2*	*0*	*0*
* Oligomenorrhea*	*9*	*5*	*4*	*0*
Dysmenorrhea	17 (32.7)	7 (33.3)	8 (34.8)	2 (25.0)

According to the Marshall and Tanner’s staging of puberty [17], the median of breast, pubic, and axillary hair stages were 4, 3, and 3, respectively in JSLE that was comparable with JIA and JDM cases. The median of the age at the puberty onset was 12 in JSLE; similarly, it was 12 in JIA and JDM girls. There were 3 JSLE, 2 JIA, and 1 JDM patients with delayed puberty. No difference was observed between the studied groups in the puberty features (Table 3).

### Hormonal profile and ultrasound features of ARD

Low levels of FSH, LH, and E2 were detected in 7, 6, and 2 cases, respectively, but serum prolactin was elevated in 2 cases of all ARDs. However, the median of FSH, LH, E2, and prolactin were comparable in all studied groups. The median of the US diameters of the endometrium, right, and left ovarian volumes was similar in the three diseases (Table 4), but US depicted infantile uterus and ovaries in 4 cases, 3 of them were JSLE and 1 had chronic JDM course.

**Table 4:** Disease activity, damage indices, hormonal assessment, ultrasound evaluation, and treatment lines in relation to menstrual cycle among cases

	Adolescent girls Median (Minimum to Maximum					
	JSLE (n = 21)		JIA (n = 23)		JDM (n = 8)	
	Menstrual abnormalities (n = 10)	Normal cycle (n = 11)	Menstrual abnormalities (n = 4)	Normal cycle (n = 19)	Menstrual abnormalities (n = 1)	Normal cycle (n = 7)
Disease duration (years)	2.5 (0.2-5)	2 (1–5)	5.5 (2–8)	3 (1–10)	5.5 (5.5–5.5)	6 (4–7)
Age of menarche	13 (0–15)	12 (11–13)	13 (11–14)	12 (11–14)	0 (0–0)	12 (11–13)
Age of puberty onset	13 (0–15) $	12 (11–13) $	13 (11–14)	12 (11–14)	0 (0–0)	12 (11–13)
Disease activity and damage indices						
Activity index/MYOACT	7 (4–25)	6 (2–20)	1 (0–2)	4 (0–33)	30 (30–30)	8 (0–12)
Damage index/MITAX	2 (0–4) $	0 (0–2) $	4 (4–4)	2 (1-2.5)	30 (30–30)	4 (0–7)
Hormonal assessment						
Estradiol (pg/mL)	28.1 (11.5–30.5)	28.7 (20.2–43.2)	30.0 (8.3–40.4)	27.6 (22.6–42.4)	19.9 (19.9–19.9)	23.7 (20.4–28.5)
Follicular stimulating hormone (mIU/L)	4.4 (2.8–8.3)	5.6 (3.8–9.3)	5.4 (2-8.3)	5.8 (2.7–8.2)	0.8 (0.8–0.8)	4.8 (3.5–8.5)
Luteinizing hormone (mIU/L)	4.5 (0.8–14.7)	5.3 (1.5–17)	3.1 (0.2–5.9)	5.0 (1.4–15)	0.1 (0.1–0.1)	4.5 (3-7.7)
Prolactin (ng/mL)	12.7 (1.5–20)	17.0 (5–22)	20.6 (13.3–29)	14.3 (1.6–27.4)	5.5 (5.5–5.5)	11 (4.8–22.8)
U/S evaluation						
Right ovarian volumes (cm3)	9 (4–11)	8 (6–11)	6 (6–11)	7 (5–11)	4 (4–4)	7 (5–7)
Left ovarian volumes (cm3)	6 (4–13)	7 (6–12)	9 (6–11)	6 (5–12)	4 (4–4)	7 (5–7)
Diameter of the endometrium (cm)	7 (4–11)	6 (6–11)	8 (6–12)	6 (5–11)	4 (4–4)	6 (6–6)
Cumulative dose of steroid (gm) *@	6 (3.6–30) $	4.3 (0.2–14) $	0.5 (0-3.6)	0.5 (0–10)	20.8 (20.8–20.8)	0.05 (0.02 to 3.6)
Other treatment lines N (%)	10 (100.0)	11 (100.0)	4 (100.0)	19 (100.0)	1 (100.0)	6 (85.7)
Cyclophosphamide use N (%)	4 (40)	0 (0.0)	0 (0.0)	0 (0.0)	0 (0.0)	0 (0.0)
Cumulative dose of cyclophosphamide (gm)	6 (6–6)	0 (0.0)	0 (0.0)	0 (0.0)	0 (0.0)	0 (0.0)

Activity index for JSLE: SLEDAI-2 K, JADAS-10 for JIA. Damage index for JSLE: SLICC/ACR, JAD for JIA. Other treatment lines: for JSLE (hydroxycholoroquine in all cases and azathioprine (19 cases) or mycophenolate mofetil (3 cases)), for JIA (methotrexate in all cases that combined with biological therapy in 3 children), while for JDM (methotrexate used in 7 patients)

$: significant difference between those with menstrual abnormalities and those with normal cycle

*: Significant difference between JSLE and JIA, *@*: Significant difference between JIA and JDM

### JSLE girls with menstrual abnormalities versus normal cycles

The median of the disease duration of JSLE with menstrual abnormalities versus normal cycles was similar. The median of the age at menarche was also alike. Regarding the age of puberty onset, it was significantly delayed in those with irregular cycles. The median of SLICC/ACR damage index was statistically higher in JSLE with abnormal menstrual cycles, but the SLEDAI-2 K did not reach statistically significant difference. The cumulative steroid dose was much higher in those with menstrual abnormalities versus regular cycles (p < 0.05) that was statistically differ than JIA or JDM cases. No significant differences were found in the other lines, CYC use, or its cumulative dose in JSLE patients with menstrual abnormalities versus regular cycles (Table 4).

The median of FSH, LH, E2, and prolactin were alike in all groups without a statistical significant difference. Similarly, the US diameters of the endometrium and ovarian volumes were comparable in the two groups (Table 4).

Apart from the onset of puberty, no statistical correlation was observed between the age of the menarche of JSLE cases and the disease duration, SLEDAI-2 K, and the cumulative dose of steroid (Table 5).

**Table 5:** Correlation between the age of menarche, disease parameters, and the cumulative doses of steroid

	JSLE		JIA		JDM	
	Age of menarche					
	r	*p*-value	r	*p*-value	r	*p*-value
Disease duration	-0.383	0.087	-0.041	0.854	-0.723	0.043*
Disease activity index	0.373	0.106	-0.257	0.324	-0.744	0.034*
Onset of puberty	0.847	< 0.001*	0.955	< 0.001*	0.710	0.049*
Cumulative dose of steroid	0.175	0.447	0.145	0.531	-0.579	0.132

Disease activity SLEDAI-2 K (for JSLE), JADAS-10 (for JIA), MYOCAT (for JDM)

* P values < 0.05 is considered statistically significant

### JIA with menstrual abnormalities versus normal cycles

The median of the disease duration, age at menarche, and age of puberty onset were similar in both subgroups. Also, the median of disease activity and damage indices were not statistically significant between those with abnormal menstrual cycles and regular ones. No significant differences were observed in the cumulative steroid dose or methotrexate use in JIA cases. The median of FSH, LH, E2, and prolactin were similar in both JIA subgroups. Likewise, the US features did not differ between the two groups (Table 4). No statistical correlation was detected between the age of menarche of JIA cases and the disease duration, JADAS-10, and steroid use except for the puberty onset (Table 5).

### JDM with menstrual abnormalities versus normal cycles

No significant differences were found between JDM cases with menstrual abnormalities and those with regular cycles regarding the demographic features, age of menarche, onset of puberty, disease assessment indices, management lines, hormonal profile, or the US findings. However, the cumulative steroid dose was statistically higher in JDM compared to JIA patients (Table 4). A statistical correlation was observed between the age of menarche of JDM cases and the studied parameters, but it was not correlated to the cumulative dose of steroid (Table 5).

### Regression analysis for menstrual abnormalities prediction in JSLE patients

In the logistic regression model, the significant predictor for menstrual abnormalities in JSLE was SLICC/ACR damage index (≥ 1; p = 0.021). However, the disease duration and cumulative dose of steroid were not detected as independent risk factors for menstrual irregularities on regression analysis (Table 6).

**Table 6:** Predictors of menstrual abnormalities among JSLE adolescent girls

Independent predictors	Unadjusted			adjusted		
	Odds ratio	95%CI	*p*-value	Odds ratio	95%CI	*p*-value
Disease duration < 2 (r) ≥ 2	1.00 1.3	0.221–7.084	> 0.99	1.00 0.671	0.11–20.98	0.768
Cumulative dose of steroid < 3.4 (r) ≥ 3.4	1.00 12.6	0.586-270.989	0.090	1.00 1.14	0.88–1.47	0.999
SLICC/ACR Damage index < 1 (r) ≥ 1	1.00 24	2.06-279.62	0.008	1.00 19.88	1.58–364	0.021*

These cut-off points based on the ROC curve, r: reference group, OR: Odds ratio, CI: Confidence Intervals, SLICC/ACR: Systemic Lupus International Collaborating Clinics/ACR damage index

* P values < 0.05 is considered statistically significant

## Discussion

There are few descriptions of menstrual abnormalities in adolescents with ARDs in the medical literature. In our study, we performed a comprehensive assessment of the impact of the three major ARDs on the ovarian function and puberty. Our analysis revealed that JSLE cases had a higher rate of menstrual abnormalities more than JIA or JDM girls. The damage index was statistically higher in JSLE with abnormal menstrual cycles, similarly were the cumulative steroid dose. However, no difference was observed between JIA or JDM subgroups. The most significant predictor for menstrual abnormalities in JSLE was the SLICC/ACR damage index as inferred from the regression analysis.

Ten patients with JSLE (47.6%) had menstrual abnormalities, whereas only four JIA and 1 JDM girls had these alterations without significant difference between the three groups. Despite the scarcity of the juvenile studies, some authors had reported that menstrual abnormalities in pediatric patients range between 23 and 63% of studied cohorts [3, 6, 16, 17, 19, 20]. A previous adult study confirmed this finding in up to 50% of lupus cases [21]. Primary amenorrhoea was observed in 3 of our JSLE patients (14.2%) and 1 JDM girl. The percentage of SLE girls with amenorrhea was 17% in **Medeiros et al., 2009** [16] cohort and it was 11.7% in another Brazilian group [20]. Our and **Silva et al., 2002** [3] studies reported six JSLE patients presented with dysmenorrhea.

The median of the age of menarche for patients with JSLE was similar to JIA and JDM groups; around the age of 12 years. The proportion of girls with delayed menarche was 8.5%. Preceding reports showed that in girls with JIA or JSLE, the menarche and puberty were delayed than in the healthy adolescent [22–24]. It was also observed to be delayed or absent in a high frequency (22%) among JSLE [25]. The median of the menstrual blood flow was the same in 3 groups (5 days); it was lower in patients with JSLE than healthy control in **Medeiros et al., 2009** [16] cohort.

In this study, there were 3 JSLE, 2 JIA, and 1 JDM patients with delayed puberty without a statistical difference between the studied groups. The prevalence of children with delayed puberty was 15% and sexual maturity was retarded for all types of JIA as shown by **Maher et al., 2013** [22]. On the other hand, the percentage of females with delayed puberty was 36.1% in **Rygg et al., 2012** [25] longitudinal cohort analysis. Meanwhile, another study demonstrated that the patterns of pubertal changes were similar in JSLE and control [16].

We observed that the median of SLICC/ACR damage index was statistically higher in JSLE with abnormal menstrual cycles, but the SLEDAI-2 K did not reach statistically significant difference. However, the disease activity nor damage index related to the menstrual disturbance in JIA or JDM cases. Formerly, it was noted that the medians of SLEDAI-2 K and SLICC-ACR/DI were also similar in JSLE patients with menstrual abnormalities versus normal cycles [3, 16]. While, it was demonstrated previously that the menstrual disturbances were related to the disease activity parameters [26].

The cumulative steroid dose was much higher in those with menstrual abnormalities versus regular cycles (p < 0.05) in JSLE girls that was statistically differ than JIA or JDM cases supporting the previous observations [24–26]. Nevertheless, an earlier analysis revealed that no impact of steroid use in girls with JIA concerning the age of menarche as compared to the healthy controls [23]. No association between CYC use or its cumulative dose and menstrual abnormalities was demonstrated in the previous reports [3, 16, 20] as also observed in our research. This finding may be attributed to the minor ovarian damage related to CYC use at this age category. The estimated risk of amenorrhea in females less than 25 years ranges between 0 and 11% [14, 27]. Moreover, the small sample size may impair a definitive conclusion regarding this point.

No significant differences were found in the other lines used in the 3 diseases between those with menstrual abnormalities versus normal cycles. The dose and duration of immunosuppressive agents were not related to the onset of puberty or gonadal function as stated by **El Badri et al., 2014** [24].

In our cohort, a limited number of cases exhibited alternation of hormonal profiles. The median of FSH, LH, E2, and prolactin were comparable in all studied groups and in those with menstrual disturbances or regular cycles. **Ronchezel et al., 2001** [28] showed that the serum prolactin was normal in JSLE patients. Moreover, **Silva et al., 2002** [3] confirmed that most cases with JSLE had normal hormonal profile. The presence of normal hormonal profile during the follicular phase may indicate a normal ovarian reserve.

The FSH is the most sensitive marker of ovarian function [29] and its level was comparable between those with or without menstrual abnormalities in JSLE as reported before [16]. Meanwhile, LH was found to be low in the adolescent lupus patients in **Medeiros et al., 2009** [16] study. The US diameters of the endometrium and ovarian volumes were comparable in all our groups likewise a former analysis [3].

No statistical correlation was detected between the age of the menarche of our cases and the studied parameters except for the age at puberty onset in all groups and the disease duration in JIA cohort. Despite of being similar between those with menstrual abnormalities and normal cycles in **Silva et al., 2002** [3] research, the disease duration and cumulative dose of prednisone showed a linear correlation with the age of menarche. These variables may be dependent on the delayed menarche of these cases.

The SLICC/ACR damage index was the significant predictor of menstrual abnormalities in JSLE patients rather than the cumulative steroid use or disease duration. Nevertheless, this finding should be considered with caution owing to the limited sample size of JSLE. Our study had some limitations as self-reporting of some variables as age of menarche, days of menstrual flow, the small sample size, and the lack of reference group for comparison. Therefore, a long-term study with a larger sample size is fundamental to confirm our findings.

## Conclusion

This study suggests that menstrual abnormalities is a common disturbance among adolescent girls with ARDs especially JSLE. However, the low amenorrhea frequency and normal hormonal profile in the majority of cases suggest that the most of these girls will attain adulthood with a great like hood of fertility. No statistical difference was observed between the studied groups in the menstrual irregularities or puberty features. The SLICC/ACR damage index is the main determinant for menstrual abnormalities in JSLE.

## Data Availability

All data generated during this study are in this published article.

## Abbreviations

ANA: Anti-nuclear antibody
ANAs: Antinuclear antibodies
ARD: Autoimmune rheumatic diseases
BMI: Body mass index
CBC: Complete blood count
CRP: C-reactive protein
CYC: Cyclophosphamide
E2: Estradiol
ESR: Erythrocyte sedimentation rate
EULAR/ACR: European League Against Rheumatism/American College of Rheumatology
FSH: Follicular stimulating hormone
ILAR: International League of Associations for Rheumatology
JAD: Juvenile arthritis damage index
JADAS-10: Juvenile arthritis disease activity score-10
JDM: Juvenile dermatomyositis
JIA: Juvenile idiopathic arthritis
JSLE: Juvenile systemic lupus erythematosus
LH: Luteinizing hormone
MDAAT: Myositis disease activity assessment tool
OR: Odds ratio
RF: Rheumatoid factor
SD: Standard deviation
SLEDAI-2K: SLE Disease Activity Index 2000
SLICC/ACR: Systemic Lupus International Collaborating Clinics/ American College of Rheumatology Damage Index
SPSS: Statistical Package of Social Science
